# Assessment and Early Intervention in the Neonatal Intensive Care Unit for a Preterm Infant With Williams Syndrome

**DOI:** 10.7759/cureus.72883

**Published:** 2024-11-02

**Authors:** Ermioni Adamian, Emmanouil Trevlakis, Ourania Papadopoulou, Koukou Zoi, Eugenia Trevlaki, Alexandra Hristara-Papadopoulou

**Affiliations:** 1 Department of Physiotherapy, School of Health Sciences, International Hellenic University, Thessaloniki, GRC; 2 Department of Midwifery, School of Health Sciences, International Hellenic University, Thessaloniki, GRC

**Keywords:** early intervention, genetic disorder, health professionals, neonatal care, neonatal intensive care, neonate, newborn baby, physical therapy, williams syndrome

## Abstract

Williams syndrome (WS) is a rare genetic disorder that affects multiple body systems and can lead to developmental delays. Early physical therapy intervention may improve neurodevelopment outcomes for newborns with WS. This case report evaluates the impact of physical therapy on the motor and sensory development of a preterm infant with WS following discharge from the neonatal intensive care unit (NICU). Physical therapy assessments were performed at one, three, and five months of age using the Alberta Infant Motor Scale (AIMS). Interventions were individualized to improve muscle strength, reflex integration, and sensory processing, and parents received guidance on home-based activities. Results showed significant growth in weight (from 3460 g to 4900 g) and length (from 51 cm to 60 cm), along with improvements in the head and chest circumference. Motor skills and reflexes, as indicated by AIMS assessments, also demonstrated improvement (p < 0.05). The infant's general health condition also improved, including the frequency and consistency of stools and the success of feeding. This case report demonstrates the effectiveness of physical therapy in improving the motor and sensory development of a newborn with WS syndrome. The combination of individualized techniques and a cooperative environment between health professionals and parents was key to the success of the intervention. Further research is needed to confirm these findings and to determine the long-term effects of early intervention on the development and well-being of these individuals.

## Introduction

Physical therapy plays a vital role in the treatment of preterm infants, especially those in neonatal intensive care units (NICUs), who are at a heightened risk of neurodevelopmental delays. These infants, placed in a foreign environment filled with excessive stimuli, require specialized intervention to facilitate proper development and help transition into a community setting [1-3]. Williams syndrome (WS) is a rare genetic condition affecting approximately one in 10,000 individuals, caused by a disruption in DNA that affects multiple body systems [4,5]. People with WS often face a range of developmental challenges, including congenital heart issues, cognitive impairments, and distinct physical features [4,6]. Early intervention is particularly beneficial for these children, as it can enhance motor and sensory outcomes, contributing to an improved quality of life and development [2,3].

## Case presentation

This study presents a case of a mother of a newborn with WS who experienced several pregnancy complications, including feeding problems, dental issues, and mental health challenges. The pregnancy was conceived spontaneously, and the delivery occurred vaginally at 36 weeks and two days. Fetal movements were felt starting in the sixth month of pregnancy. During routine prenatal checks, WS was diagnosed in the fetus, and close monitoring was performed until birth. The newborn was born prematurely, weighing 1880 g, with a cleft palate. Due to respiratory issues, the newborn was stabilized using a Neopuff T-piece resuscitator before being transferred to the NICU for mild respiratory distress. The infant required support for gastroesophageal reflux disease (GERD) due to an immature lower esophageal sphincter and received feeding education from a speech therapist. The cleft palate required evaluation by a pediatric surgeon for future surgical repair. While at the NICU, the newborn had reduced resistance to passive movements, severe back tone, and pathological head support. During the two-month stay in the NICU, the infant's weight increased by 1580 g, length increased by 5 cm, head circumference increased by 4.5 cm, and chest circumference increased by 1.5 cm. Physical therapy interventions were performed by a hospital physical therapist two to three times per week, focusing on improving muscle tone, motor skills, and reflex integration through neurodevelopmental techniques. Upon NICU discharge, parents were given instructions for the newborn's care, including regular pediatric monitoring, vaccination, vitamin D and iron supplementation (D-Fe), specialized feeding recommendations (using A2 formula), pediatric gastroenterology assessment, and continued physical therapy, along with follow-up appointments.

**Figure 1:**
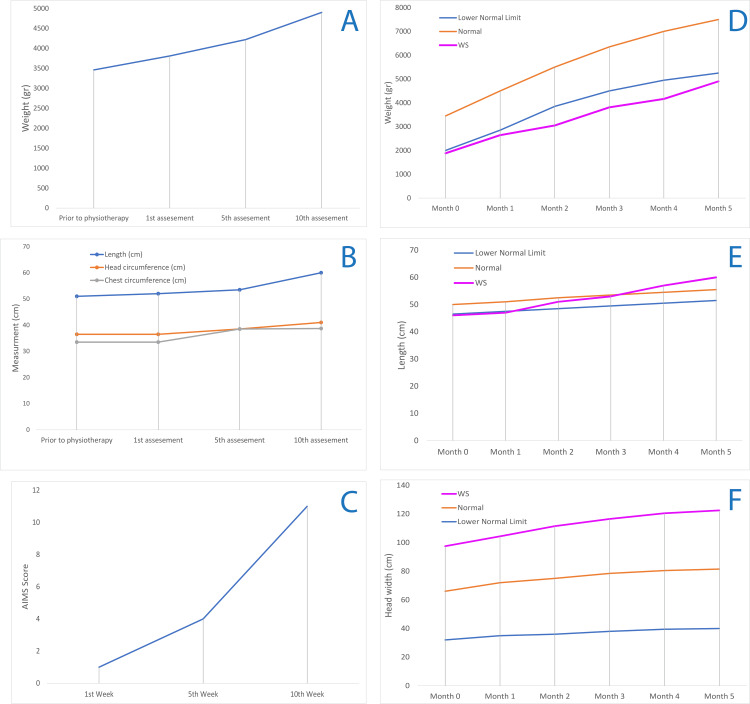
Newborn recorded data. A. Weight (gr) from the birth of the premature infant until before the physio-therapeutic intervention, B. Length, head, and chest circumference from the birth of the premature infant until before the physio-therapeutic intervention. C, D, E. Weight, length, and head circumference compared to the normal and lower normal growth limits.

After NICU discharge, a total of 20 therapeutic interventions and 10 evaluations were conducted at the researcher's pediatric physical therapy clinic over a period of three months. Visits occurred twice weekly for therapy and once weekly for evaluations, each lasting 45 minutes. These sessions focused on improving muscle strength, reflex integration, and sensory processing, with regular assessments using the Alberta Infant Motor Scale (AIMS). The newborn underwent an extended stay in the NICU for approximately two months, during which biometric data, including weight, height, head circumference, chest circumference, temperature, oxygen saturation, and heart rate, were recorded every 15 days by the hospital's physiotherapist (see Table 1 and Figure 1A-1E). These data showed consistent improvements in growth parameters, with physical therapy possibly contributing to enhanced motor development and reflex responses [7].

**Table 1:** Biometric data of the newborn from birth up to prior to physiotherapy AIMS: Alberta Infant Motor Scale

	Birth	Discharge	Prior to physiotherapy
Weight (g)	1880	3050	3460
Length (cm)	46	47	51
Head circumference (cm)	31,5	32,4	36,5
Chest circumference (cm)	32	32,6	33,5
Blood Pressure (mmHg))	-	80-40	80-40
Ο_2_ (%)	97	98	97
Temperature (°C )	36,9	36,8	36,8
Pulse (beats per minute)	141	140	139
AIMS	-	-	1

Methodology

The purpose of this clinical case report was to investigate the role of assessment in the treatment of premature newborns with WS by pediatric physical therapists, both in the NICU and a pediatric physical therapy clinic. This study aimed to examine the impact of early intervention by pediatric physical therapists on the health outcomes of premature newborns, specifically addressing motor and sensory development. The study was conducted in accordance with ethical standards and received approval from the International Hellenic University Bioethics Research Committee on 30/03/2022, ensuring that all procedures met the required ethical guidelines for research involving human subjects. The parents also signed an informed consent agreement, acknowledging their understanding of the study’s purpose, procedures, and their rights, thereby providing formal authorization for their infant's participation.

Participants

This report focuses on a single preterm infant born at 36 weeks gestation with a confirmed diagnosis of WS. The infant was identified as small for gestational age (SGA) according to the Fenton growth curves.

Assessment Tools and Role of Assessment

Assessment played a critical role in both the identification of developmental challenges and the tailoring of interventions for the infant. During the NICU stay and subsequent follow-up period, a range of assessment tools were utilized to guide treatment. The AIMS [8] was used to evaluate motor development, providing a standardized measure of progress in gross motor skills. In addition, the evaluation of primitive reflexes, such as the Asymmetric Tonic Neck Reflex (ATNR), was crucial in identifying reflex integration and developmental status.

These assessments helped the physical therapists monitor the infant’s progress and adjust treatment goals accordingly. Regular assessments were conducted at one, three, and five months of corrected age, allowing for a dynamic and responsive intervention plan. The data collected from these assessments were used to modify physical therapy techniques, ensuring the interventions remained individualized and effective. The role of assessment was not only to track developmental milestones but also to identify areas requiring additional support, thereby optimizing the intervention strategy.

Intervention

During the first two months in the NICU, the infant underwent physical therapy sessions performed by a hospital-based pediatric physical therapist, two to three times per week. The interventions aimed to address reduced resistance to passive movements, severe back tone, and pathological head support. The specific techniques employed included neurodevelopmental techniques to improve muscle tone, reflex integration, and sensory processing.

Upon discharge, parents received detailed care instructions including recommendations for regular pediatric monitoring, vaccinations, supplementation with vitamins (D and Fe), specialized feeding guidance using A2 formula, assessments by a pediatric gastroenterologist, and ongoing physical therapy.

Post-discharge, a total of 20 physical therapy interventions and 10 evaluations were conducted over three months at a pediatric physical therapy clinic, with twice-weekly therapy sessions and weekly evaluations lasting 45 minutes each. The infant's age at the 1st assessment was three months (corrected age of four months), and at the 10th and final assessment, it was five months (corrected age of six months). These sessions focused on enhancing muscle strength, reflex integration, and motor development, using individualized interventions tailored to the infant's specific needs.

Data Collection

During the NICU stay, the weight, length, head circumference, chest circumference, temperature, oxygen saturation, and heart rate, were recorded every 15 days by the hospital physiotherapist (see Table 1 and Figure 1A-1E). After discharge, the same parameters were monitored at each physical therapy visit at the clinic.

Data Analysis

Data analysis was performed using IBM SPSS (version 26.0, Armonk, NY), with Microsoft Excel 2013 (Microsdoft Corp., USA) employed for graph creation. Statistical significance was set at p < 0.05 for all analyses.

Treatment

**Figure 2:**
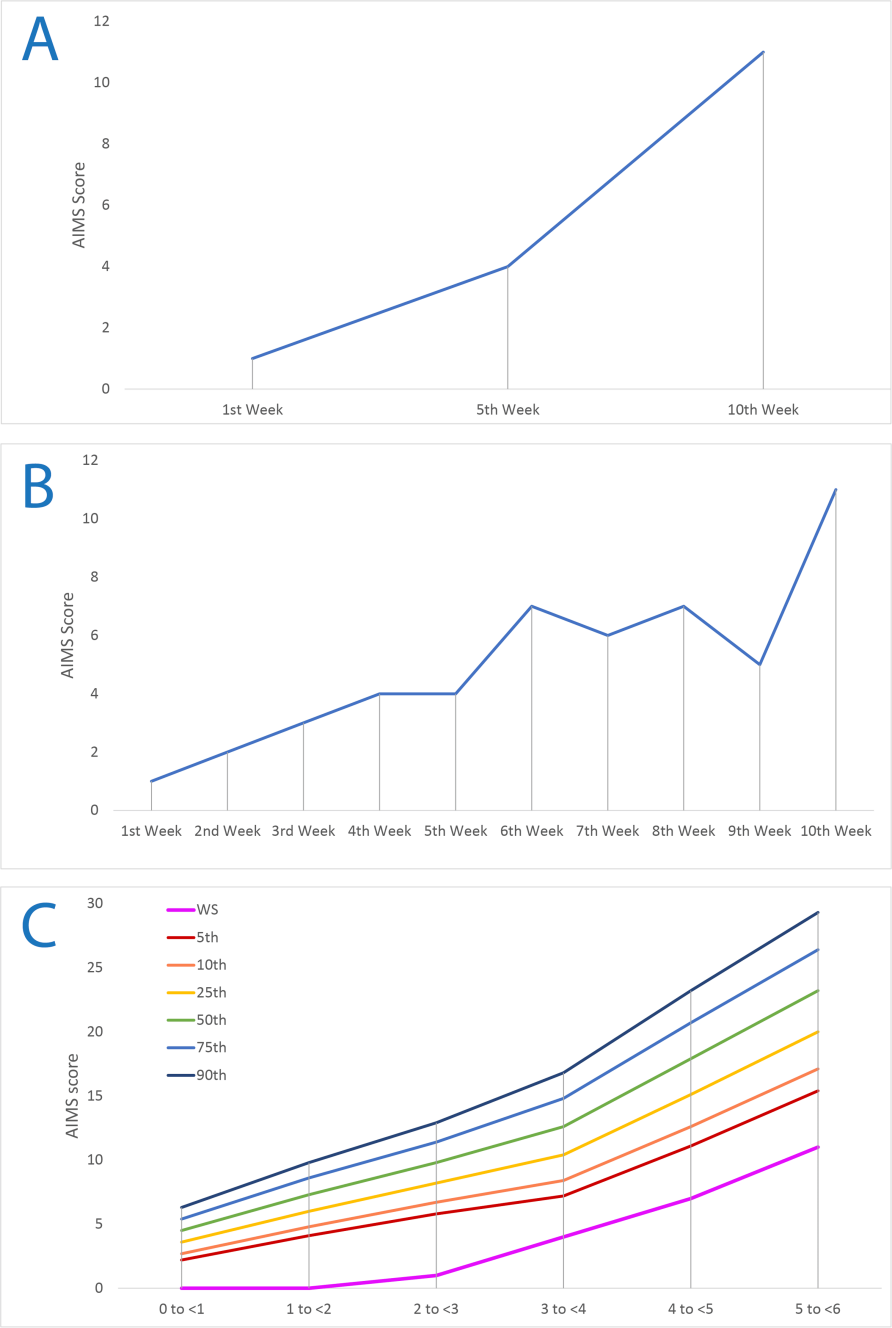
A. AIMS score at the first, fifth, and 10th week. B. AIMS score across the intervention. C. AIMS quintiles of the newborn together with the normal growth quintiles. AIMS: Alberta Infant Motor Scale

**Figure 3:**
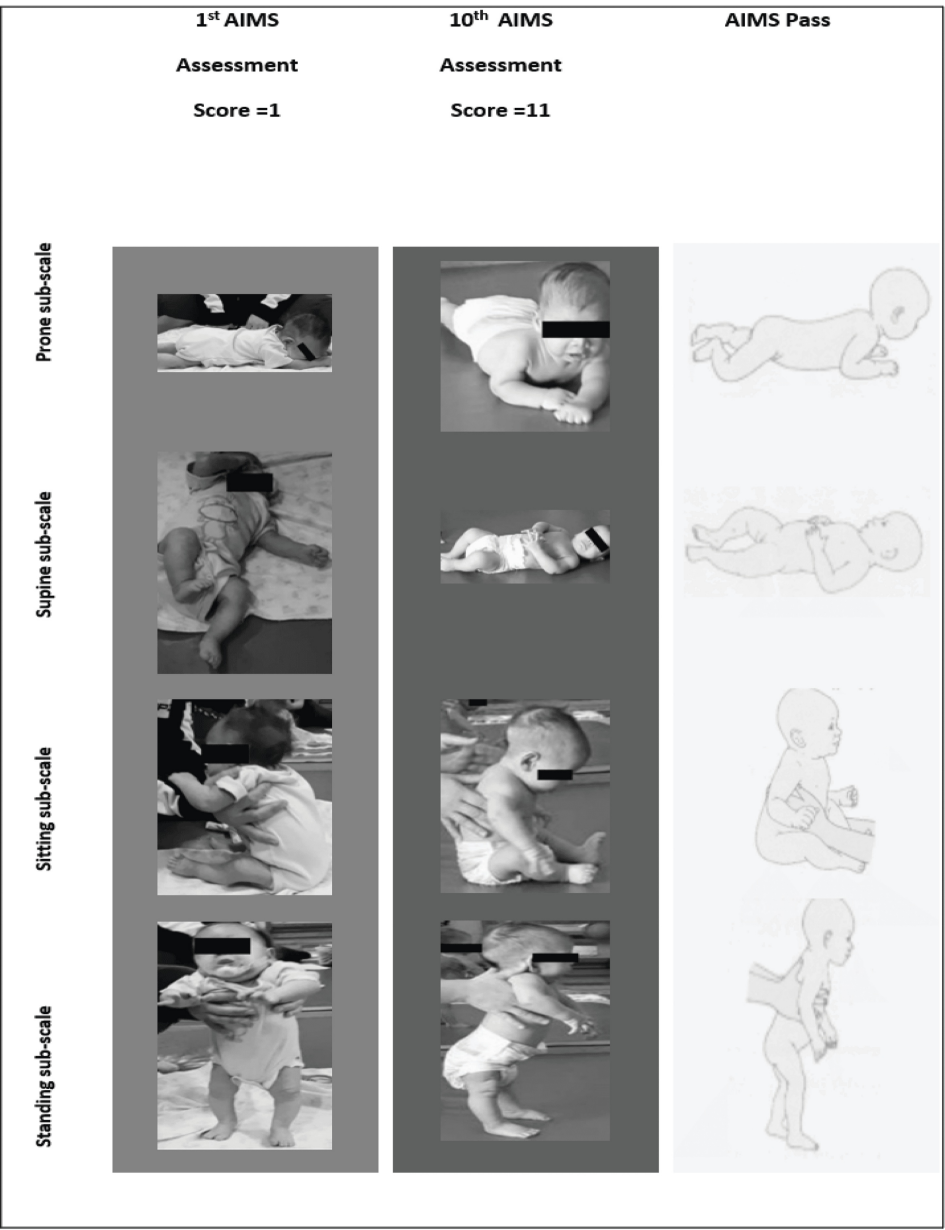
Comparison of the first and 10th assessment AIMS scores compared to the pass on the AIMS AIMS: Alberta Infant Motor Scale

Table 2 illustrates the measurement time points for the premature neonate prior to physical therapy and during consecutive assessments. Table 3 provides an overall summary of the evaluations of reflexes for the infant between two and five months of age. Table 4 presents the assessment of the physical therapy intervention according to the AIMS. Figure 2 illustrates the quintile scores of the infant on the AIMS, while Figure 3 contrasts the first assessment against the last assessment in terms of normal scores.

**Table 2:** Biometric data of the newborn during physiotherapy AIMS: Alberta Infant Motor Scale

	Prior to physiotherapy	First assessment	Fifth assessment	10th assessment	Mean	SD
Weight (gr)	3460	3810	4220	4900	4006	±995.64
Length (cm)	51	52	53.5	60	54.15	±3.49
Head circumference (cm)	36.5	36.5	38.5	41	37.87	±4.13
Chest circumference (cm)	33.5	33.5	38.5	38.7	35.67	±2.45
Blood pressure (mmHg)	80-40	80-40	100-50	100-55	90-55	-
Ο_2_ (%)	97	98	98	99	98	±0.99
Temperature (^o^C)	36.8	36.9	36.8	36.9	37	±0.78
Pulse (beats per minute)	139	140	139	117	133.75	±3.51
AIMS	1	1	4	11	4.25	±3.76

**Table 3:** Reflex assessment

Reflexes/reactions	Two months	Three months	Five months
Asymmetric tonic neck reflex, (ATNR)	Present	More intense	More intense
Symmetrical Tonic Neck Reflex (STNR)	-	Weakened	Weakened
Spinal galant reflex	Weakened	-	-
Stepping reflex	Not present	-	-
Τonic labyrinthine reflex (TLR)	Not present	Not present	Not present
Head righting reflex (HRR)	Not present	-	-
Landau reflex	-	Weakened	Weakened
Blinking reflex		Not present	Not present
Labyrinthine-head righting reflex		Not present	Not present

**Table 4:** Assessment of motor development using the AIMS AIMS: Alberta Infant Motor Scale

Assessment of motor development using the AIMS	
Torso and head	Backstroke movements are more controlled. Head control has improved. The trunk is more active, with better use of abdominal and back muscles. The pelvis is also more active during anteroposterior tilts. In the supine position, the infant momentarily tries to rest their chin on their sternum using neck flexors, indicating improvement in the labyrinthine and visual systems. The trunk in the supine position is more symmetrical. Rolling from the supine to side or prone positions is usually induced by turning the head. Stimulation of the cervical response causes the entire trunk to turn to the side. In the prone position, the infant extends their head and can maintain this position for a longer period of time with visual or auditory stimuli. The infant can stay in the prone position for longer periods and has better support on their forearms. Support on the forearms in the prone position is momentary and occurs when the head is bent.
Upper and lower extremities	The infant is using their upper and lower limbs more effectively. Shoulder girdle and upper limb synergy is slightly improved. Control of the shoulder girdle is not sufficient to allow the chest to rise and maintain the head in a neutral position. Lower limb movements usually mimic upper limb movements and alternate between flexion and extension. Flexion and extension of the lower limbs promote anteroposterior mobility of the pelvis. Asymmetrical positions of the lower and upper extremities cause weight shifts to one side. The lower limbs try to be symmetrically extended, but there is not a full range of extension in the hip, knee, and ankle joints.
Interacting with objects and people	Although hand control is limited, the infant tries to reach objects with their hands and bring them to their face or chest, not always successfully. Hand-eye synergy has improved in the midline position. There has been an increase in the visual field. The infant tries to grab toys and bring them to their mouth. The infant tracks people and objects laughs at faces and is more communicative. Their sensorimotor and sensory development has improved.

Biometrics

The infant's weight increased from 3460 g at the start of the treatment to 4900 g by the end of the NICU stay, with a mean of 4006 g (±995.64). The growth indices for weight showed an improvement that brought the infant close to the lower normal limits for the fifth month of life (5250 g, Figure 1C). The length also increased significantly from 51 cm to 60 cm, with a mean of 54.15 cm (±3.49), which was above the minimum normal limit and near normal levels by the fifth month. Head circumference increased from 36.5 cm to 41 cm, with a mean of 37.87 cm (±4.13), exceeding the lower normal limit and almost reaching typical levels by the final assessment (Figure 1E). The chest circumference showed a smaller increase, from 33.5 cm to 38.7 cm, with a mean of 35.67 cm (±2.45), indicating statistical significance in growth (Figure 1B). The recorded parameters were also used to assess the infant's vital signs and general health condition. Blood pressure was monitored at each stage, starting from 80/40 mmHg and eventually increasing to 100/55 mmHg. Oxygen saturation (SpO_2_) was measured at 97-99%, showing stable respiratory health. Temperature was maintained within the range of 36.8°C to 37°C. Heart rate was recorded consistently at 117-140 bpm (Figure 1B).

Reflexes and Reactions

At two months of age, the infant displayed the ATNR, which affected symmetry and restricted head movement by locking the gaze to the tilted side. The infant also demonstrated challenges in bringing their hands to the mouth, with the right hand being released less effectively. The spinal galant reflex was present but weakened, likely due to hypotonia. Walking reflexes, body-head reflexes, and the spinal extensor reflex were absent at this stage. At three months, the ATNR persisted and became more pronounced on the left side. The symmetric tonic neck reflex (STNR) and Landau reflex were present but impaired. The spinal stretch reflex was absent, while both the visual corrective reaction and the labyrinthine corrective reaction were noted. These reactions played a crucial role in facilitating head righting and maintaining balance when the infant was in different positions. By five months, the ATNR was still present, with increased intensity on the left side, while the STNR and Landau reaction continued to show signs of weakness. The spinal stretch reflex remained absent. However, the visual and labyrinthine corrective reactions were present, contributing to improved head and body coordination. The persistence of these primitive reflexes and the absence of expected responses such as the tonic labyrinthine reflex (TLR) and head righting reflex (HRR) indicated developmental delays that required continuous monitoring. Table 3 presents all the information.

Assessment of motor development using the AIMS

At each AIMS assessment, significant changes were noted in both the quantity and quality of the infant's movements. For instance, improvements in head control, trunk stability, and upper and lower limb movements were observed. The infant demonstrated increased control over backstroke movements, with improved head support and trunk activity. The active use of abdominal and back muscles during anteroposterior tilts indicated progress in the integration of the labyrinthine and visual systems.

Initially, the infant struggled with symmetrical movements of the upper and lower extremities, which led to difficulties in weight shifting and positioning. However, as therapy progressed, the infant's ability to interact with objects improved. Hand-eye coordination in the midline position became more effective, and the infant began to reach for toys and bring them closer to the face and chest, although not always successfully.

The AIMS scores for the infant were recorded and compared across assessments (see Figure 2). The infant initially fell into a low-scoring quintile, which indicated a motor deficit and the need for a targeted intervention plan. Over time, however, a statistically significant (p-value < 0.05) improvement was observed, both in terms of overall motor function and in the ability to achieve and maintain postures relevant to gross motor milestones. The quintile position also shifted to indicate improvement, though the infant's scores remained below the typical range for age-matched peers.

Infant Feeding

The amount of milk consumed by the infant increased by 10-20 ml after discharge from the NICU, and there was a noticeable increase in feeding frequency. Initially, the infant had difficulty maintaining a rhythm in sucking movements during breastfeeding attempts, but these issues significantly improved with time. Due to the cleft palate, breastfeeding was not successful, and bottle feeding became the preferred method. The infant was fed using a bottle with a specially designed nipple for cleft palate, which helped minimize milk spillage and allowed for more efficient feeding.

Despite these adaptations, there was still some milk diffusion in the oral cavity during feeding, and the infant had difficulty bringing their lips together, resulting in the mouth remaining constantly open.

To further support feeding success, parents were also advised to stop feeding if they observed any pathological movements of the tongue and jaw or paradoxical swallowing of milk. The cleft palate’s severity, along with the associated oral-motor challenges, required ongoing assessment and specialized feeding interventions to ensure proper growth and nutritional intake.

Figure 3 has a visual comparison for the first and 10th assessment AIMS scores for the infant.

Instructions for parents

Upon discharge from the NICU, parents were provided with detailed instructions to support the ongoing care and development of their infant. These instructions included recommendations for positioning, feeding, physical therapy exercises, and environmental considerations that could help optimize the infant's health outcomes.

The feeding regimen included the use of specialized feeding equipment, such as a bottle with a cleft palate nipple, to support efficient and effective milk intake. Parents were also instructed to observe the infant closely for signs of difficulty during feeding, such as pathological movements of the tongue or jaw, or paradoxical swallowing. If any such signs were observed, feeding was to be paused to avoid complications.

Parents were also instructed on how to perform the prescribed physical therapy exercises at home to complement the sessions conducted at the clinic. These exercises focused on improving muscle tone, reflex integration, and promoting symmetrical movement patterns. Parents were provided with guidance on selecting appropriate toys to encourage visual tracking, reaching, and other sensorimotor activities essential for the infant’s developmental progress. The parents did not report any issues following the instructions.

## Discussion

In this case report, the physical therapy interventions administered to the infant were associated with significant motor improvements and increased growth parameters, including weight, length, head circumference, and chest circumference. It is important to note that while motor improvements can be attributed to physical therapy interventions [8,9], the increase in weight, length, and head circumference cannot be solely attributed to these interventions, as growth in these parameters is generally expected during infancy. Instead, the role of physical therapy in enhancing the infant's motor development may have indirectly supported overall growth by improving feeding efficacy and reducing feeding-related challenges.

Previous studies have highlighted the benefits of early physical therapy intervention in supporting the motor and sensory development of premature newborns in the NICU. Similar conclusions were reached in a case study of a female infant with WS who received early intervention at seven months of age, which demonstrated significant motor improvements after intervention [9]. The findings in our case are consistent with the established literature emphasizing the value of early intervention for infants with complex medical conditions, particularly in improving motor skills through targeted therapies [1,2,10-11].

Assessment of motor skills using AIMS provided an objective measure to monitor changes over time and guide interventions [12-14]. AIMS scores for this infant improved significantly over the intervention period, demonstrating progress in gross motor skills, such as head control, trunk stability, and reaching movements. The evaluation of primitive reflexes, such as the ATNR, was also instrumental in understanding the infant's developmental trajectory and identifying areas needing focused intervention.

Our findings highlight the importance of individualized techniques and the use of reliable assessment tools, such as AIMS and reflex evaluations, in guiding treatment and tracking the progress of premature newborns. While the current case is a promising example of the role of physical therapy in supporting motor development, it is important to acknowledge the limitations of attributing growth entirely to physical therapy interventions.

## Conclusions

The present case report confirms the importance and necessity of early physical therapy intervention for premature newborns in the NICU. The findings highlight the value of individualized techniques and the use of reliable assessment tools, such as the Evaluation of Primitive Reflexes and Reactions and the AIMS, in promoting motor and sensory development. The study also emphasizes the benefit of providing comprehensive information to parents, both during pregnancy and after birth, as well as the importance of cooperation between an interdisciplinary medical team.

In conclusion, early intervention, effective use of reliable assessment tools, and collaboration within an interdisciplinary medical team, alongside comprehensive support for parents, collectively contribute to the improved neurodevelopmental outcomes of premature newborns in the NICU.

Future studies should aim to evaluate the long-term effects of physical therapy interventions, starting from the NICU through post-discharge, to understand better the evolution of motor, sensory, and mental development in premature infants.
